# Development and Evaluation of Tannic Acid-Coated Nanosuspension for Enhancing Oral Bioavailability of Curcumin

**DOI:** 10.3390/pharmaceutics13091460

**Published:** 2021-09-13

**Authors:** Hyeonmin Lee, Jun-Bae Bang, Young-Guk Na, Jae-Young Lee, Cheong-Weon Cho, Jong-Suep Baek, Hong-Ki Lee

**Affiliations:** 1 College of Pharmacy, Chungnam National University, 99 Daehak-ro, Yuseong-gu, Daejeon 34134, Korea; gusals2218@naver.com (H.L.); bangjb95@hotmail.com (J.-B.B.); youngguk@cnu.ac.kr (Y.-G.N.); jaeyoung@cnu.ac.kr (J.-Y.L.); 2 Institute of Drug Research and Development, Chungnam National University, 99 Daehak-ro, Yuseong-gu, Daejeon 34134, Korea; 3 Department of Bio-Health Convergence, Kangwon National University, Chuncheon 24341, Korea; 4 Department of Herbal Medicine Resource, Kangwon National University, 346 Hwangjo-gil, Dogye-eup, Samcheok-si 25949, Korea; 5 Animal Model Research Group, Jeonbuk Branch, Korea Institute of Toxicology (KIT), Jeongeup 53212, Korea

**Keywords:** curcumin, nanosuspension, tannic acid, mucoadhesion, statistical design

## Abstract

Curcumin (CUR) has been used in the treatment of various diseases such as cough, fever, skin disease, and infection because of various biological benefits such as anti-inflammatory, antiviral, antibacterial, and antitumor activity. However, CUR is a BCS class 4 group and has a limitation of low bioavailability due to low solubility and permeability. Therefore, the purpose of this study is to prepare a nanosuspension (NSP) loaded with CUR (CUR-NSP) using a statistical design approach to improve the oral bioavailability of CUR, and then to develop CUR-NSP coated with tannic acid to increase the mucoadhesion in the GI tract. Firstly, the optimized CUR-NSP, composed of sodium dodecyl sulfate (SDS) and polyvinylpyrrolidone/vinyl acetate (PVP/VA), was modified with tannic acid (TA). The particle size and polydispersity index of the formulation measured by laser scattering analyzer were 127.7 ± 1.3 nm and 0.227 ± 0.010, respectively. In addition, the precipitation in distilled water (DW) was 1.52 ± 0.58%. Using a differential scanning calorimeter and X-ray diffraction analysis, the stable amorphous form of CUR was confirmed in the formulation, and it was confirmed that CUR-NSP formulation was coated with TA through a Fourier transform-infrared spectroscopy. In the mucoadhesion assay using the turbidity, it was confirmed that TA-CUR-NSP had higher affinity for mucus than CUR-NSP under all pH conditions. This means that the absorption of CUR can be improved by increasing the retention time in the GI tract of the formulation. In addition, the drug release profile showed more than 80% release, and in the cellular uptake study, the absorption of the formulation (TA-CUR-NSP) containing TA acting as an inhibitor of P-gp was increased by 1.6-fold. In the evaluation of antioxidant activity, the SOD activity of TA-CUR-NSP was remarkably high due to TA, which improves cellular uptake and has antioxidant activity. In the pharmacokinetic evaluation, the maximum drug plasma concentration of the TA-coated NSP formulation was 7.2-fold higher than that of the pure drug. In all experiments, it was confirmed that the TA-CUR-NSP is a promising approach to overcome the low oral bioavailability of CUR.

## 1. Introduction

Turmeric has been used for thousands of years in traditional Asian medicine due to its health benefits, and much research is currently being conducted on its potential in pharmaceuticals, foods, and cosmetic [1]. Curcumin (CUR) is the most used in turmeric for the treatment of a variety of diseases and a highly biologically active ingredient [2,3]. In addition, CUR is known to have various biological advantages such as antifungal [4], antiviral [5], antibacterial [6], anti-inflammatory [7], antioxidant [8], and antitumor [9] activity.

Despite these many advantages, CUR has significant limitations due to its low bioavailability due to low intrinsic activity, low absorption by the low water solubility, high metabolism, inactivity of metabolites, and/or rapid removal from the body [10]. Therefore, there is a need for an appropriate formulation capable of increasing the bioavailability of CUR.

Nanosuspension (NSP) rapidly increases the saturation solubility and dissolution rate by reducing the particle size to the nanometer range and increasing the surface area by using a suitable polymer and surfactant [11]. As a result, it has been considered one of the most promising approaches to improving the bioavailability of insoluble active pharmaceutical ingredients [12,13]. Besides, NSP has advantages such as a simple manufacturing process and easy scale-up [14].

Mucoadhesive drug delivery system interacts with the epithelial surface and mucus layer of the mucosa to enhance the residence time of the dosage form at the absorption site and promote close contact at the absorption surface to improve the therapeutic performance of the drug [15].

Tannic acid (TA) is one of the abundant polyphenols and is generally recognized as safe (GRAS) by the FDA [16]. In addition, some studies show that TA increases adhesion when combined with polymers such as polyvinylpyrrolidone (PVP) or poly(ethylene glycol) (PEG) [16,17]. Moreover, according to the literature, TA has posed as a promising pharmaceutical candidate due to its potential anticancer activities [18]. TA involves in the several oncological signaling pathways such as JAK/STAT, mTOR, BCL-2, SOX-2, VEGF/VEGFR, and CXCL12/CXCR4 axes [18]. Thus, nanotechnology using a TA would improve not only the mucoadhesive function of NSP, but also the anticancer activity. Considering the combinational beneficial effects of TA with CUR, we aim to develop the TA-coated CUR-NSP (TA-CUR-NSP) for the improvement of the solubility, mucoadhesion, and gastrointestinal permeability of CUR.

Polymeric nanoparticles are surrounded by a polymeric shell or polymeric network, while the nanosuspension is the stabilized colloidal dispersions in the presence of stabilizers (polymer or surfactant, or both). In this study, CUR was stabilized in the presence of surfactants and polymers; moreover, CUR-NSP was developed using an anti-solvent precipitation method. The antisolvent precipitation method has been known as one of the bottom-up techniques for the development of nanosuspension. Various nanosuspension formulations by the antisolvent precipitation method have been introduced and developed in literature [19,20,21]. In this study, sodium dodecyl sulfate (SDS) and polyvinylpyrrolidone/vinyl acetate (PVP/VA) were used as stabilizers. SDS is an anionic surfactant and FDA-approved excipient in oral delivery, primarily functioning as a dissolution agent and a stabilizer, and it may also act as a protease inhibitor [22]. PVP/VA is a polymer with increased physical stability by partial replacement of hygroscopic VP with hydrophobic VA [23]. CUR-NSP developed by the anti-solvent precipitation method was coated with TA. TA-CUR-NSP was assumed to improve oral absorption and bioavailability by improving the solubility, mucoadhesion, and gastrointestinal permeability of CUR.

## 2. Materials and Methods

### 2.1. Materials

**Figure 1:**
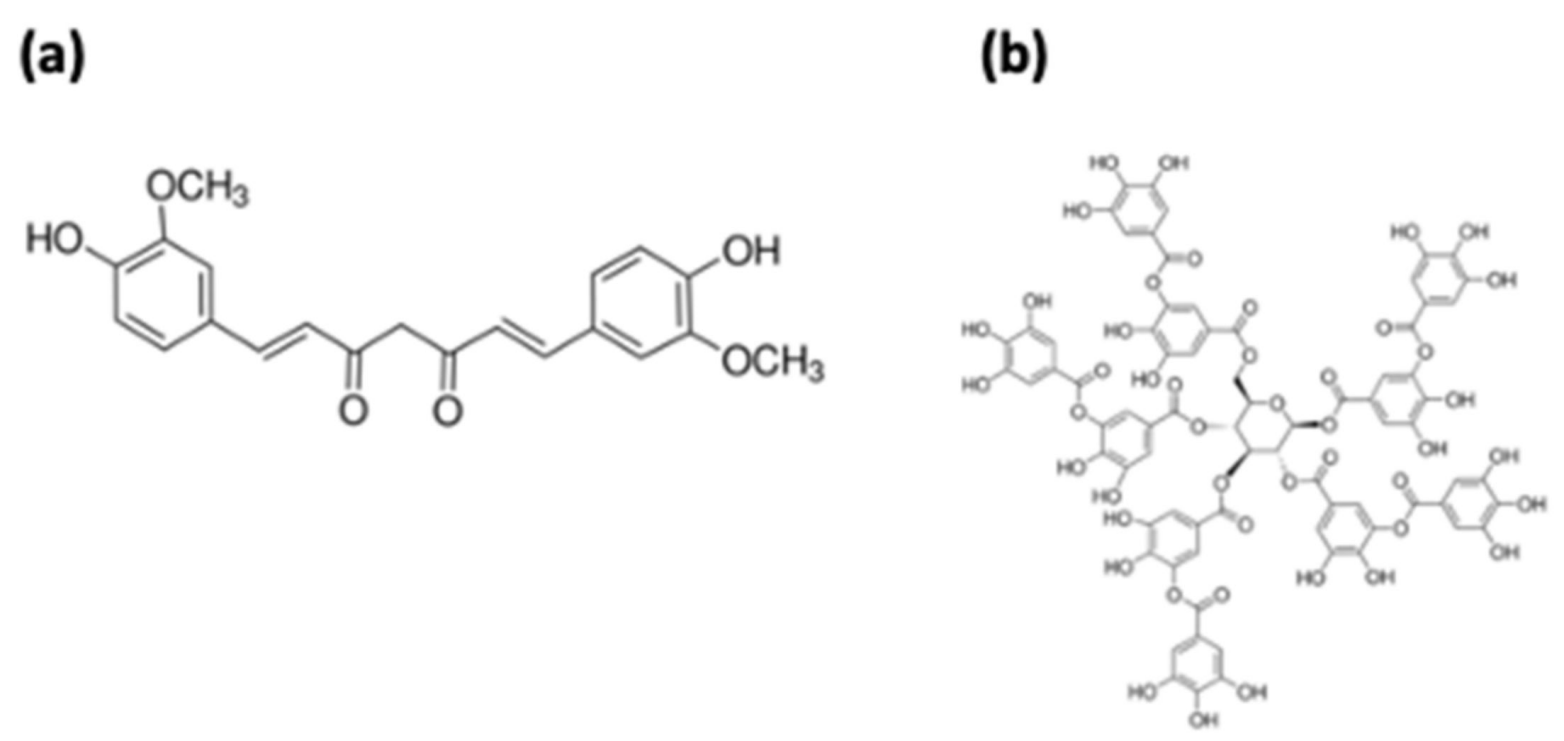
Chemical structure of CUR (**a**) and TA (**b**).

CUR (Turmeric, Diferuloylmethane, impurity >65%, Figure 1a), tannic acid (TA, ACS reagent, Figure 1b), SDS, Hexadecyltrimethylammonium bromide (CTAB), Hydroxypropyl-β-cyclodextrin (HPβCD, molecular weight (MW) 1396 da), D-α-Tocopherol polyethylene glycol 1000 succinate (TPGS), polyvinyl alcohol (PVA, MW 30,000–70,000), and sodium carboxymethylcellulose (Na CMC) were purchased from Sigma-Aldrich (St. Louis, MO, USA). According to the literature, the turmeric CUR consists of various ingredients, in particular demethoxycurcumin [24]. Therefore, the coexisting ingredient such as demethoxycurcumin could be contributed to the efficacy of CUR. However, in this study, the turmeric CUR was used as the control and we focused to compare the efficacy between the turmeric CUR and CUR-NSP. Solutol HS15, Cremophor EL, Cremophor RH40, Cremophor ELP, poloxamer 407, and poloxamer 188 were acquired from BASF (Ludwigshafen, Germany). Hydroxypropyl cellulose (HPC, L grade) was obtained from Nippon soda Co., Ltd. (Tokyo, Japan). Hydroxypropyl methylcellulose (HPMC, METOLOSE 60SH-50) was provided by Shin-Etsu Chemical CO., Ltd. (Tokyo, Japan). PVP (K-30) and PVP/VA (S-630) were purchased from Ashland Inc. (Covington, KY, USA). Tween 20 and Tween 80 were purchased from Samchun Chemical (Pyungtaek, Korea). Micro BCATM Protein Assay Kit and Dulbecco’s Modified Eagle’s Medium were obtained from Thermo Fisher Scientific (Rockford, IL, USA). ProEXTM CETi lysis buffer (TLP-121.1) was obtained from TransLab (Daejeon, Korea). Caco-2 cell (heterogeneous human epithelial colorectal adenocarcinoma) was purchased from Korean Cell Line Bank (Seoul, Korea). Polyvinylidene fluoride (PVDF, 0.45 μm) filter was purchased from Whatman^®^ (Maidstone, UK).

### 2.2. HPLC Analysis

Shimadzu LC-2030c 3D (Shimadzu Corporation, Kyoto, Japan) was used for CUR analysis [25]. The HPLC column was a Kinetex^®^ C18 100 Å column (250 mm × 4.6 mm id, 5 μm) (Phenomenex, Torrance, CA) and the mobile phase consisted of 0.1% trifluoroacetic acid in distilled water (DW) adjusted to pH 3.0 with ammonia solution (A) and acetonitrile (B) (55:45% *v*/*v*). The temperature of the column was kept at 40 °C, and the flow rate was 0.8 mL/min. The UV detection wavelength was 425 nm and the injection volume was 20 μL.

### 2.3. Screening of Surfactants and Polymers

To screen the various surfactants and polymers, equilibrium solubility of CUR was evaluated. In brief, an excess CUR was added into 1 mL of different surfactant solutions (1% *w*/*v*). Then, they were rotated for 72 h using an angle rotator (Model AG; Fine PCR, Gunpo, Korea). After the rotation, they were centrifuged at 15,000 *g* for 10 min, and the supernatants were collected. The supernatant was filtered using a 0.45 μm polyvinylidene fluoride (PVDF) filter. Filtrates were diluted with methanol and injected into HPLC.

### 2.4. Preparation of CUR-NSP

The antisolvent precipitation method was used for the preparation of CUR-NSP. The solvent phase was used in 1 mL of acetone with 30 mg curcumin dissolved. The aqueous phase was used 10 mL of DW included with surfactant (5 mg) and polymer (200 mg). The 1 mL of the solvent phase was added to the aqueous phase using a syringe prepared with no. 22 needle gauge while stirring at 750 rpm and kept stirring overnight to evaporate all the organic solvents at room temperature. Moreover, we stirred them in order to minimize the coalescence. The blank-NSP was prepared without CUR in the same method. For the characterization studies, CUR-NSP was freeze-dried using lyophilizer (FD-1000, EYELA, Tokyo, Japan). The freeze-drying step comprised of freezing at −70 °C for 4 h using Ultra-Low Temperature Freezer (MDF-U52Z, SANYO Electric Biomedical Co., Ltd., Osaka, Japan) and drying at −57 °C for 48 h using lyophilizer. At drying step, constant pressure of 9.5 Pa was applied. In this study, the CUR-NSP was freeze-dried in the absence of the cryoprotector. Even though the CUR-NSP was freeze-dried without the cryoprotector, the physicochemical properties such as the size and PDI were maintained.

### 2.5. Optimization of CUR-NSP

#### 2.5.1. Design of Experiments

For the optimization of CUR-NSP, the Box–Behnken design (response surface method, RSM) was used, which was carried out using Design Expert^®^ 12 (Stat-Ease Inc., Minneapolis, MN, USA). RSM is the experimental design, which is commonly used for the optimization of formulations and processes [26]. The advantage of the Box–Behnken design is that it saves money and time because it requires fewer experiments compared with other designs, including central composite designs. The three factors and three response designs were considered for the optimization (Table 1). The amount of SDS (X_1_, mg) and PVP/VA (X_2_, mg) and the volume of aqueous phase (X_3_, mL) were set as factors, and the range of factors were 0–5 mg, 150–250 mg, and 5–15 mL, respectively. The particle size (Y_1_), PDI (Y_2_), and precipitation (Y_3_) of CUR-NSP were set as responses, and the responses of 17 experiments were fitted to models including linear model, cubic model, quadratic model, special cubic, or quartic model. The appropriate model was suggested by comparing statistical parameters.

#### 2.5.2. Particle Size (Y_1_) and Polydispersity Index (Y_2_)

To evaluate the particle size and PDI of CUR-NSP, an electrophoretic laser scattering (ELS) analyzer (ELS-Z2; Otsuka Electronics, Osaka, Japan) was used. In brief, the samples were diluted with DW and placed into cuvette. Then, the sample placed in the cuvette was monitored by ELS analyzer. Measurement was conducted for 50 times for each sample.

#### 2.5.3. Precipitation (Y_3_)

Through precipitation measurement, it was confirmed that CUR-NSP forms a homogeneous and stable nano-sized suspension. Simply, 10 mL of CUR-NSP was filtered using a 5 μm pore size filter (Advantec No. 2 filter paper; Toyo Roshi Kaisha, Ltd., Tokyo, Japan). Then, they were diluted with methanol and injected into HPLC. The precipitation (%) was calculated as the below equation:

(1) $$Precipitaion\;\left(\%\right)=100\times \left(\frac{C_{non-filter}-C_{filter}}{C_{non-filter}}\right),$$

where *C_filter_* is the CUR concentration in filtered CUR-NSP, and *C_non-filter_* is the CUR concentration in non-filtered CUR-NSP.

### 2.6. Preparation of TA-CUR-NSP

**Scheme 1:**
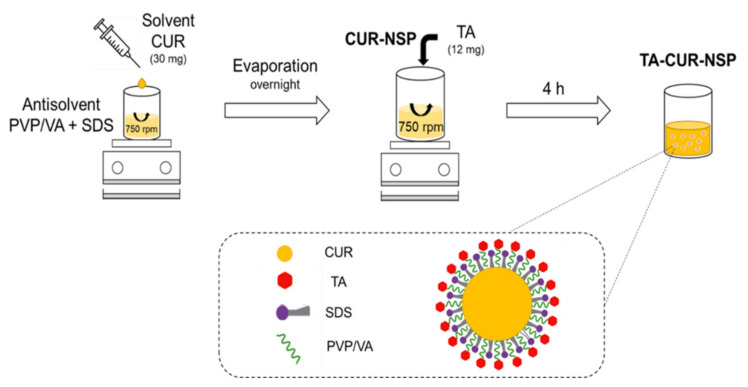
Preparation process of TA-CUR-NSP.

TA-CUR-NSP was prepared by coating the TA on CUR-NSP. In brief, 30 mg curcumin was dissolved in the solvent phase (1 mL of acetone). The aqueous phase was prepared by dissolving the PVP/VA (150 mg) and SDS (3 mg) in 12 mL of DW. Then, 1 mL of the solvent phase was added to the aqueous phase using a syringe prepared with no. 22 needle gauge while stirring at 750 rpm and kept stirring overnight to evaporate all the organic solvents at room temperature. To ameliorate mucoadhesion in the GI tract, CUR-NSP was coated with TA. After the organic solvents were evaporated, 12 mg of TA was added into CUR-NSP by stirring for 4 h (Scheme 1).

### 2.7. Characterization of Optimized CUR-NSP and TA-CUR-NSP

#### 2.7.1. Differential Scanning Calorimetry

To evaluate the change in crystalline form, differential scanning calorimetry (DSC) analysis was carried out with DSC N-650 model (Scinco, Seoul, Korea). The samples (2 mg; CUR, PVP/VA, SDS, CUR-NSP, and physical mixture, TA, TA-CUR-NSP) were put in aluminum pans. Then, the samples were heated with a heating rate of 20 °C/min under nitrogen flow (30–300 °C), and the DSC thermograms were recorded.

#### 2.7.2. Powder X-ray Diffraction

An X-ray diffractometer (D/MAX-2200 Ultima/PC, Rigaku Corporation, Tokyo, Japan) was used to measure the powder X-ray diffractions (PXRD) of the samples. The scan range (2θ) was ranged from 4 to 60° and the scanning speed was set as 0.02°/s.

#### 2.7.3. Fourier Transform-Infrared Spectroscopy

Interactions between the components were measured with an ALPHAP FT-IR spectrometer (Bruker Optics Inc., Billerica, MA, USA), and Fourier transform-infrared spectroscopy (FT-IR) spectra were investigated. The spectra were plotted from 4000 cm^−1^ to 600 cm^−1^.

### 2.8. In Vitro Release Study

In vitro CUR release from TA-CUR-NSP compared to CUR-NSP and physical mixture were studied. TA-CUR-NSP, CUR-NSP, and physical mixture (4 mL; equivalent to 10 mg of CUR) were placed in the dialysis bags (100 kDa MWCO) and incubated in 75 mL of PBS with constant stirring (100 rpm). Sink conditions were met by supplementing all release mediums with 0.5% (*w*/*v*) SDS. Moreover, 0.5 mL of the samples were collected at regular intervals and replaced with the same amount of fresh medium. The collected samples were filtered through a 0.22 µm syringe filter and analyzed by HPLC. The release experiments were performed in triplicate.

### 2.9. Mucoadhesion Assay

The interaction between NSPs and mucus layer was investigated according to the modified method [27]. Mucin suspensions were prepared at a concentration of 1% (*w*/*v*) by suspending and continuously stirring in pH buffers (pH 1.2, 4.0, 6.8, and DW), overnight. Then, they were incubated at 37 °C for one night. After that, they were centrifuged at 1000 *g* for 20 min, and the supernatants were collected and diluted to 0.5% (*v*/*v*). Diluents were incubated with the same volumes of CUR-NSP or TA-CUR-NSP at 37 °C, 150 rpm. Moreover, at constant time intervals (0, 0.5, 1, 2, and 4 h), the turbidity of all the samples including mucin suspensions and formulations mixed with pH buffers without mucin were measured by UV spectroscopy at 500 nm. All measurements were performed in triplicate.

### 2.10. Cell Studies

#### 2.10.1. Cell Culture

Caco-2 cell was cultured in Dulbecco’s Modified Eagle’s Medium with 10% fetal bovine serum and 1% penicillin/streptomycin, maintaining at 37 °C with 5% CO_2_.

#### 2.10.2. Cytotoxicity Study

The MTT assay was performed to evaluate the cytotoxicity of the formulation against Caco-2 cells. Briefly, cells (3 × 10^4^ cells/well, 100 µL) were seeded into 96-well plates and incubated for 24 h. Then, CUR-formulations (pure CUR, CUR-NSP, and TA-CUR-NSP corresponding to 0.01–50 µg/mL CUR) and blank formulations (blank-NSP and TA-blank-NSP corresponding to 1–100 µg/mL formulation) were treated, and they were incubated for 24 h. After the incubation, MTT solution was dispersed into each well (30 µL/well, 5 mg/mL), and the plate was incubated for 4 h. After the removal of media, 200 µL of DMSO was added to each well, and the absorbance was measured using a microplate reader (Sunrise; Tecan Group Ltd., Männedorf, Switzerland) at 565 nm. Cell viability was calculated by below equation:

(2) $$Cell\;viability\;\left(\%\right)=\frac{{Abs}_{sample}}{{Abs}_{control}}\times 100,$$

where *Abs_sample_* and *Abs_control_* are the absorbance of the sample treated and untreated cells, respectively.

#### 2.10.3. Cellular Uptake Study

For quantitative study, Caco-2 cells were seeded into 6-well plate at 1 × 10^6^ cells/well. After the cells reached confluence, each plate was incubated with 30 µg/mL of pure CUR, CUR-NSP, or TA-CUR-NSP at 37 °C. After 4 h incubation, the sample solutions in plates were removed, and the plates were washed twice with cold PBS. After that, 0.5 mL of ProEXTM CETi lysis buffer (TLP-121.1, TransLab, Daejeon, Korea) was added into each well for cell lysis. For the extraction of CUR from the cells, 0.5 mL of acetonitrile was added into the lysis solution. The mixture was centrifuged at 15,000 *g* for 10 min. Then, the supernatant was measured by HPLC. The cellular uptake of CUR from CUR-NSP and TA-CUR-NSP was normalized with the amount of protein using BCA assay.

Visualization of cellular uptake of TA-CUR-NSP was observed by performing fluorescence observation. Caco-2 cells (1 × 10^3^ cells/well, 3 mL) were distributed into 6-well plates and incubated at 37 °C for 24 h. Pure CUR and NSPs (corresponding to 30 μg/mL CUR) were diluted with cell medium containing 1% DMSO (*v*/*v*) and treated to each well. (Control treated with 1% DMSO (*v*/*v*) in cell medium.) After incubation for 4 h, the plate was washed twice using cold PBS and 4% of formaldehyde was added for 5 min. Thereafter, 4′,6-diamidino-2-phenylidone (DAPI) solution was treated for 5 min to stain the cell nuclei. Then, the wells were washed twice with cold PBS and the cells were observed using EVOSTM M5000 imaging system (Thermo Fisher Scientific, Waltham, MA, USA).

### 2.11. Antioxidant Activity

To evaluate the optimal condition for inducing the oxidative stress, Caco-2 cells were seeded at 1 × 10^6^ cells/well in a 6-well plate. In the experiments purposed at evaluating the antioxidant activity of NSPs, Caco-2 cells were incubated for 24 h with pure CUR, CUR-NSP, or TA-CUR-NSP (corresponding to 30 μg/mL CUR) and 12 μg/mL of TA. For the control group, the medium was added and incubated. Then, a medium containing sample solution was removed, and the cells were treated with H_2_O_2_ (500 µM) for 24 h. ELISA kit (Thermo Fisher Scientific Inc., Rockford, IL, USA) was used for the quantitative estimation of superoxide dismutase (SOD) levels in the cell using a microplate leader. Each sample for the parameter was analyzed in triplicate, and optical density values were verified against a standard curve.

### 2.12. Animal Studies

#### 2.12.1. Animals

For the pharmacokinetic evaluation of formulations, male Sprague-Dawley (SD) rats, weighing 250–300 g, was used, and animals were purchased from Nara-Biotec (Seoul, Korea). Before the experiment, animals were housed in polycarbonate cages and acclimated for a week. They were housed at 22 °C and relative humidity of 55%, and water and food were supplied ad libitum. All animal experiments were conducted in accordance with the guidelines (Animal Use Guidelines) approved by Chungnam National University Institutional Animal Care and Use Committee (Daejeon, Korea).

#### 2.12.2. Pharmacokinetic Study

The rats were divided into 3 groups, and there were 5 rats in each group. The freeze-dried CUR-NSP and TA-CUR-NSP and pure CUR after being dispersed with DW were delivered to rats by oral delivery (the dose of 35 mg/kg and volume of 10 mL/kg). After the administration of formulations, the blood samples (200 µL) were collected via jugular vein at 0.17, 0.34, 0.67, 1, 1.5, 2, 4, 6, 8, 12, and 24 h. After the centrifugation, the plasma samples were collected and stored at −70 °C until HPLC analysis.

#### 2.12.3. Sample Preparation and Analytical Methods

For analyzing CUR in plasma samples, 90 μL of plasma was put into the tube, and 10 µL of internal standard (IS, 4-hydroxybenzophenone, 10 μg/mL in methanol) solution and 200 µL of methanol were mixed. Then, the samples were mixed for 5 min for 1000 rpm, and then centrifuged at 15,000 g for 10 min. The obtained supernatants were transferred to vials for analysis.

The chromatographic separation was completed on Gemini^®^ C18 110 Å column (250 mm × 4.6 mm id, 5 μm) (Phenomenex, Torrance, CA) using a Shimadzu LC-2030c 3D (Shimadzu Corporation, Kyoto, Japan). The mobile phase consisted of 1% citric acid in DW adjusted to pH 3.0 with ammonia solution (solution A) and acetonitrile (solution B) (45:55% *v*/*v*). Analysis was conducted at a flow 0.8 mL/min and the column temperature was kept at 30 °C. The UV detection wavelength was 300 and 428 nm for IS and CUR, respectively.

## 3. Results and Discussion

### 3.1. Screening of Surfactants and Polymers

Surfactants and polymers were screened to determine the appropriate excipients that could significantly increase the solubility of the CUR to form a stable NSP.

**Figure 2:**
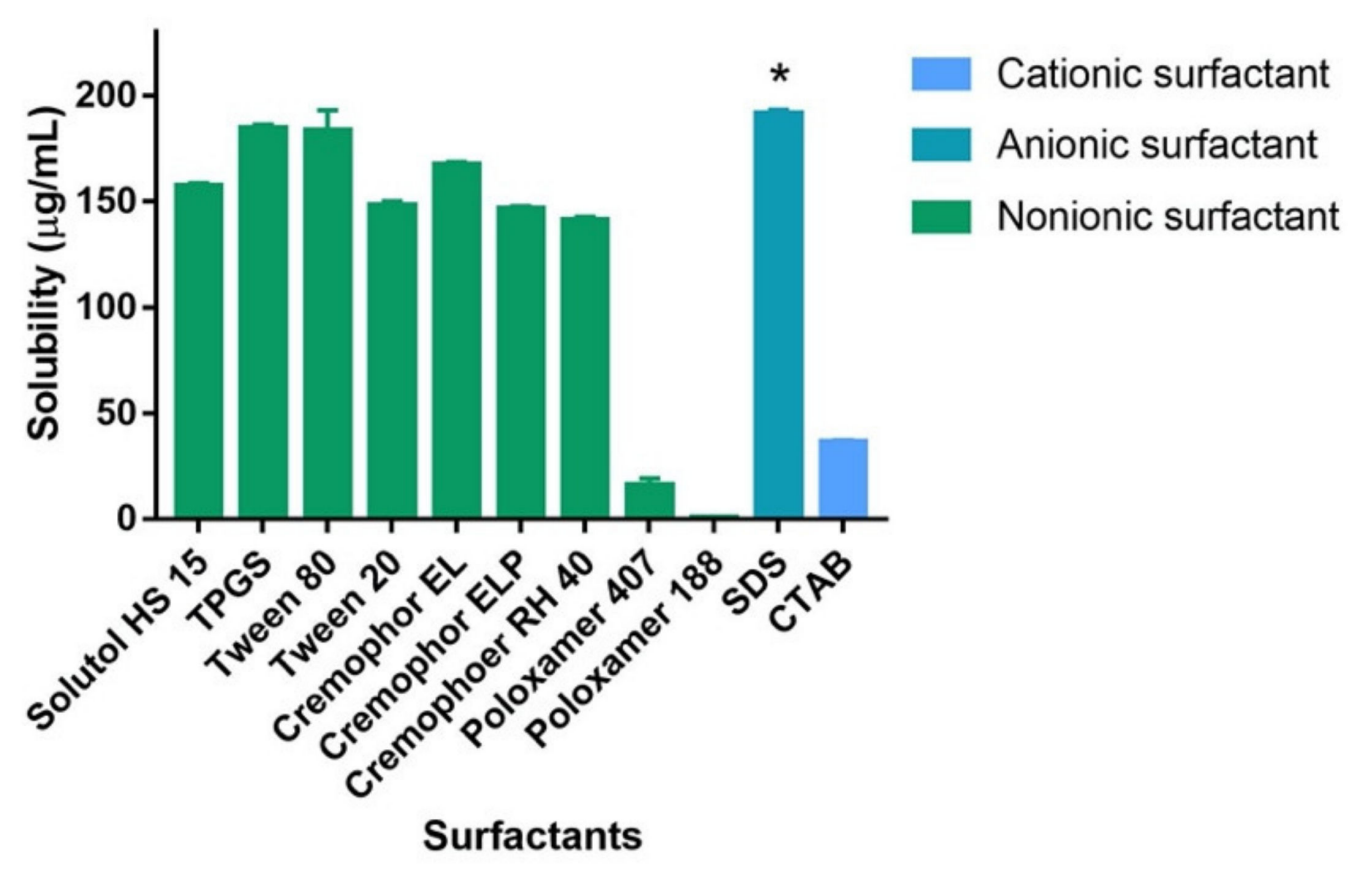
The solubility of CUR in various 1% (*w*/*v*) surfactants (hexadecyltrimethylammonium bromide, CTAB; sodium dodecyl sulfate, SDS; D-α-Tocopherol polyethylene golycol 1000 succinate, TPGS) solution for 72 h (*n* = 3). Values are expressed as the mean ± SD. (* *p* < 0.05).

The investigated surfactants were SDS, Solutol HS 15, D-α-Tocopherol polyethylene glycol 1000 succinate (TPGS), Cremophor EL, Tween 20, Tween 80, Cremophor ELP, Poloxamer 407, Cremophor RH 40, Poloxamer 188, and CTAB. Initial screening of the surfactants was performed based on the solubility of CUR in the 1% surfactant solutions. Among the various surfactants, the CUR solubility in SDS showed the highest value at 191.37 μg/mL (Figure 2). Therefore, SDS was selected as a surfactant for preparing CUR-NSP. In this study, nonionic (Solutol, TPGS, Tween, Cremophor, Poloxamer), anionic (SDS), and cationic (CTAB) surfactants were evaluated to select the stabilizer for the fabrication of CUR-NSP. According to the literature, the toxicity of surfactants generally demonstrates in order of the cationic surfactants > anionic surfactants ≥ zwitterionic surfactants > nonionic surfactants [28]. However, for the development of NSP, various factors including solubility, forming ability, and encapsulation should be considered. For example, it has been reported that an anionic surfactant (SDS) had little effect on the release rate of the drug from solid dispersions [28]. Moreover, in this study, CUR-NSP including SDS showed acceptable cytotoxicity against the intestinal epithelial cell.

**Figure 3:**
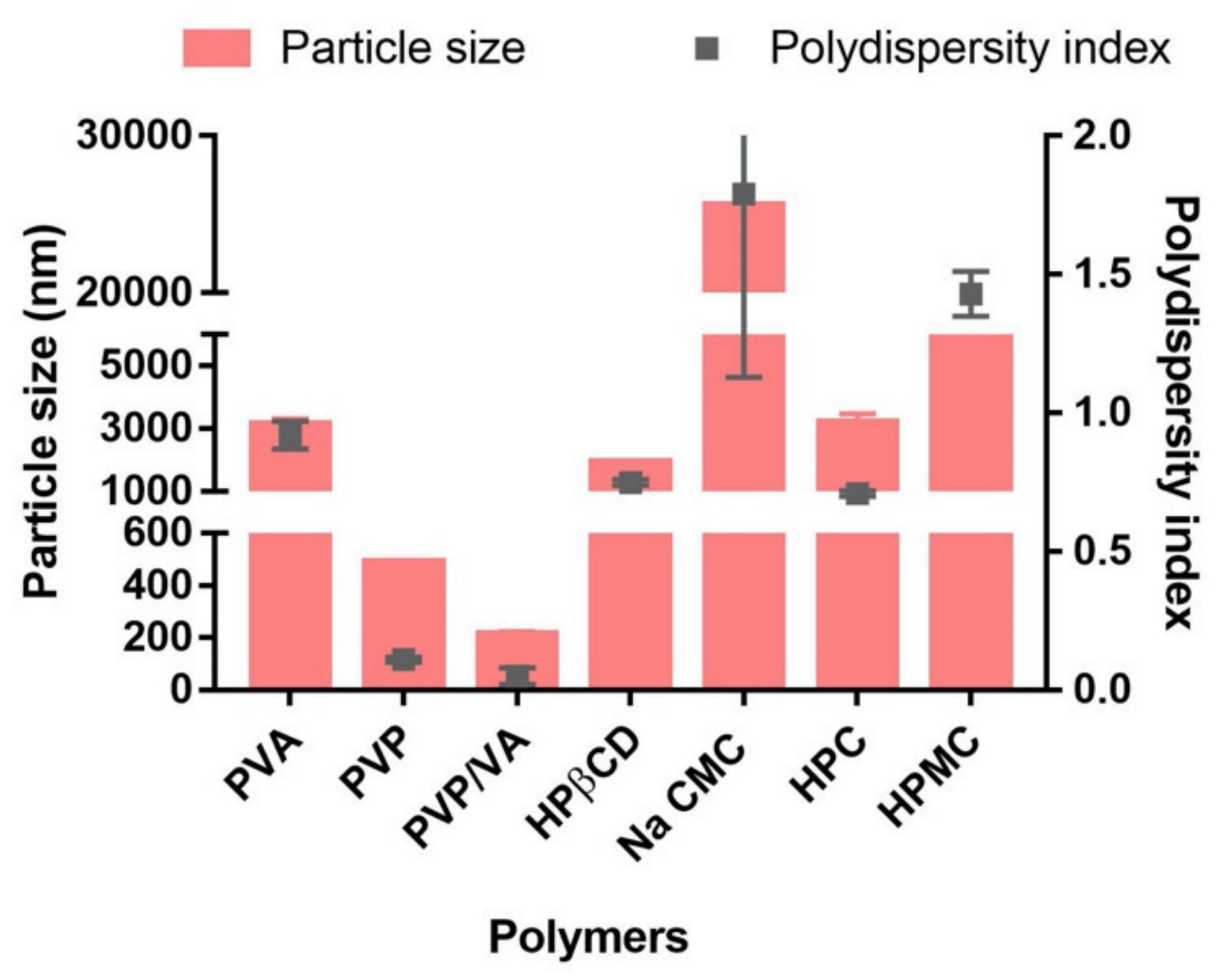
Particle size and polydispersity index prepared with various polymers (carboxymethyl cellulose sodium salt, Na CMC; hydroxypropyl-β-cyclodextrin, HPβCD; hydroxypropyl cellulose, HPC; hydroxypropyl methylcellulose, HPMC; polyvinyl alcohol, PVA; polyvinylpyrrolidone, PVP; polyvinylpyrrolidone/vinyl acetate, PVP/VA). Values are expressed as the mean ± SD (*n* = 3).

Based on the solubility study, initial NSPs were prepared with SDS and various polymers (PVP/VA, PVA, PVP, HPβCD, Na CMC, HPC, and HPMC). We evaluated the particle size and PDI of the NSP to select the proper polymer. Among the polymers used for screening, CUR-NSP prepared using PVP/VA exhibited the smallest particle size and PDI (Figure 3). Accordingly, PVP/VA was selected as a polymer that could prevent aggregation of drug particles.

### 3.2. Optimization of CUR-NSP

The three factors were set as the amount of SDS (X_1_), the amount of PVP/VA (X_2_), and the volume of the aqueous phase (X_3_), respectively. The amount of SDS (X_1_) and PVP/VA (X_2_) affects the solubility of CUR and the adequate steric repulsion to cover the entire surface of the nanoparticle, respectively [29]. Moreover, the volume of aqueous phase (X_3_) could change the viscosity of the formulation. The small volume of aqueous phase increases the viscosity of the formulation, preventing the diffusion of drug between the solvent and the antisolvent. Thus, the small volume of the aqueous phase leads to large and non-homogeneous particle sizes [30].

Y_1_ (Particle size), Y_2_ (PDI), and Y_3_ (precipitation) were selected as responses in evaluating the optimized CUR-NSP. These responses relate to the stability of nanoparticles and bioavailability. When the nanoparticle with a small particle size (Y_1_) enters GI tract, the nanoparticle could readily permeate the membrane to increase the precision of contact with the membrane [31]. Therefore, it improves the oral absorption of CUR. Narrow and homogeneous size distribution (Y_2_) of the nanoparticle could affect the stability and efficiency of CUR-NSP [32]. Furthermore, the low precipitation (Y_3_) of nanoparticle indicates that large amount of drug is encapsulated in the NSP [29].

Table 2 lists the composition of CUR-NSP and observed responses. The statistical models and parameters such as *p*-value, lack of fit *p*-value, R^2^, adjusted R^2^, and predicted R^2^ are listed in Table 3. Quadratic model was suggested for all responses. The sequential *p*-values signify that the suggested models were meaningful at the 95% confidence level and less than 0.05 in all models. To explain the suitableness between factors and responses, the lack of fit *p*-value was evaluated and > 0.05 of this value indicated the suitable model [33].

The R^2^ and adjusted R^2^ values indicate the variability between the suggested model and the experimental data [29]. In Table 3, R^2^ and adjusted R^2^ were above 0.90 and 0.87, respectively. It indicates that the suggested models well predicted the responses. Moreover, R^2^ and adjusted R^2^ showed the difference of less than 0.1; thus, the suggested model was well fitted.

**Figure 4:**
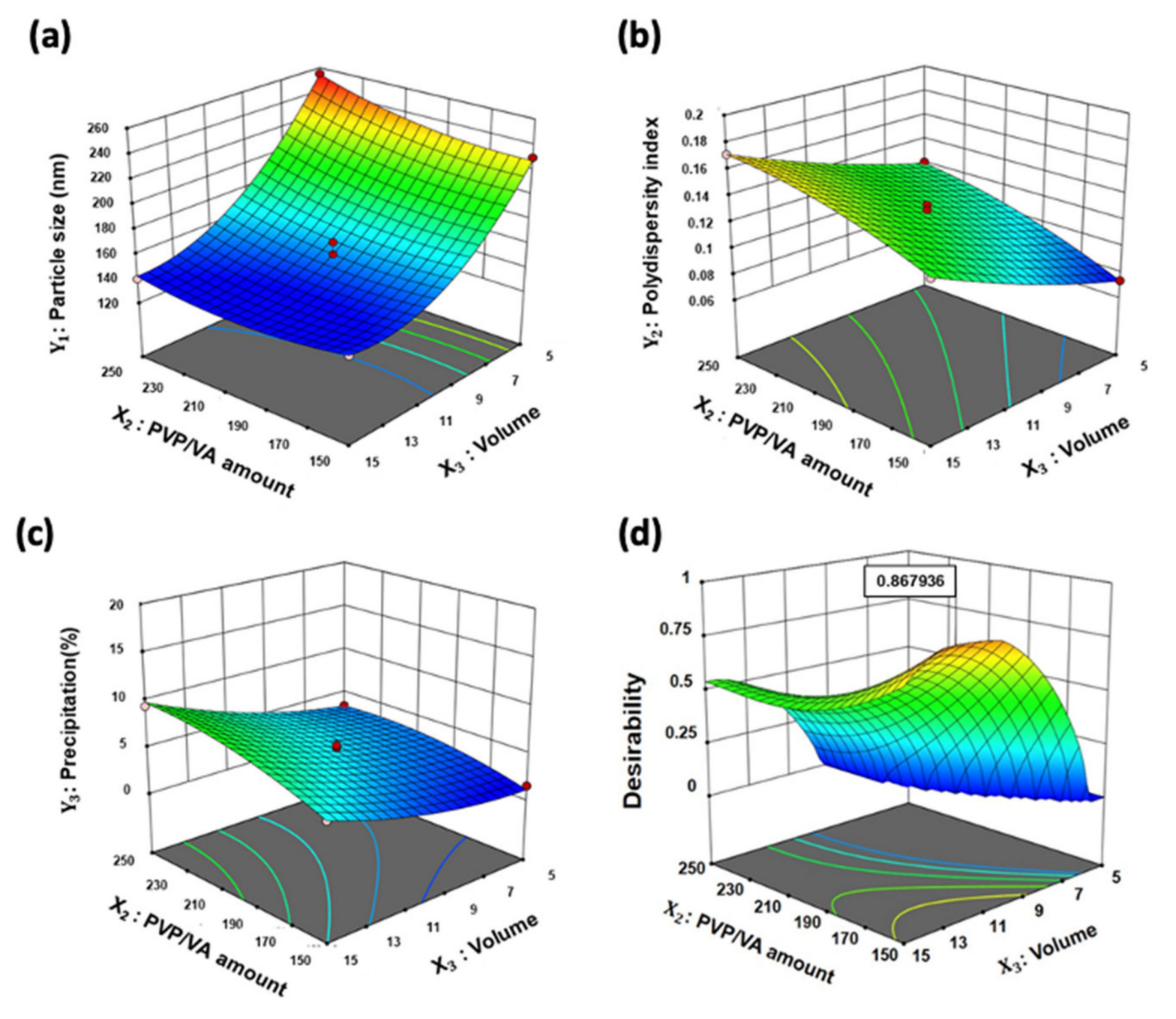
Three-dimensional response surface plots; (**a**) Y_1_: particle size; (**b**) Y_2_: polydispersity index; and (**c**) Y_3_: precipitation. (**d**) Desirability plot of numerical optimization.

The relationships between factors and responses were expressed through three-dimensional plots and coded equations. The amount of SDS (X_1_) was fixed with 3 mg because the high value of X_1_ excessively increased the solubility of CUR so that particles were not formed properly. The three-dimensional plot and actual values according to the experimental design are shown in Figure 4. The rages of Y_1_, Y_2_, and Y_3_ values were from 133.4 to 253.1 nm, 0.080 to 0.195, and 1.51 to 16.70%, respectively. In the case of Y_1_, the value increased as the value of X_2_, and the value decreased as the value of X_3_ increased, and X_3_ had the more influence on Y_1_ between X_2_ and X_3_ (Figure 4a). In the case of Y_2_, Y_2_ increased as each factor also increased (Figure 4b). For Y_3_, lower X_2_ and higher X_3_ reduce Y_3_ (Figure 4c).

To optimize the composition of CUR-NSP, a desirability plot was used, and the optimal desirability was determined. Figure 4d illustrates the desirability plot, and the optimal values of X_1_, X_2_, and X_3_ were 3, 150 mg, and 12 mL, respectively. Moreover, the predicted responses were compared to the actual values of the optimized CUR-NSP (Table 4). When the responses of the optimized CUR-NSP were evaluated, particle size (Y_1_), PDI (Y_2_), and precipitation (Y_3_) were 146.1 nm, 0.108, and 1.55%, respectively. These values were in the range of 95% CI of the predicted responses. Thus, it suggests that the optimization of CUR-NSP was successfully conducted.

### 3.3. Preparation of TA-CUR-NSP

**Figure 5:**
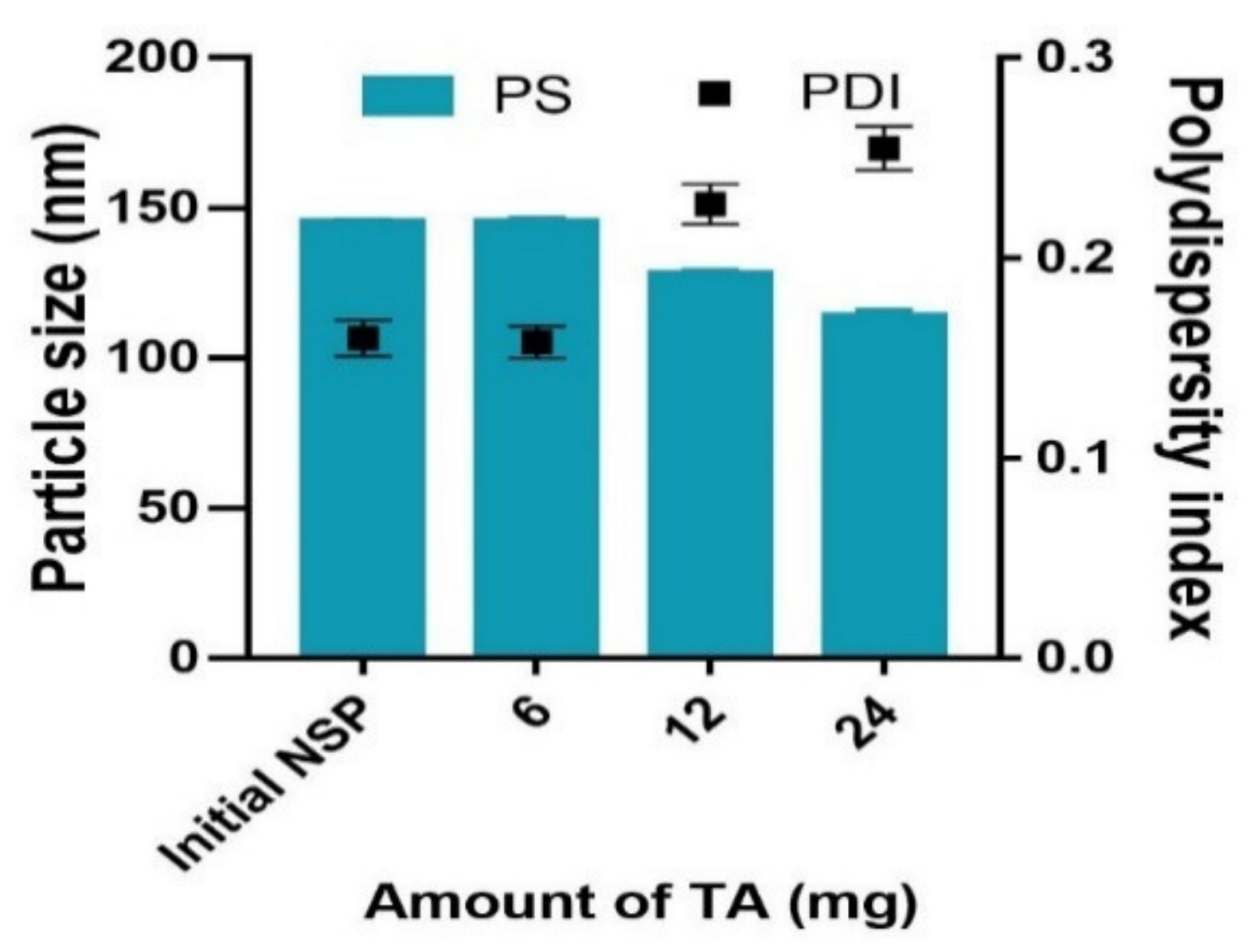
Changes in particle size and polydispersity index according to the amount of TA.

To improve mucoadhesion in the GI tract, optimized CUR-NSP was modified with TA. The particle size and PDI of CUR-NSP without TA were 145.1 ± 1.0 nm and 0.160 ± 0.009. When 6 mg of TA was added, particle size and PDI were 145.2 ± 1.1 nm and 0.158 ± 0.008. When 12 mg of TA was added, the changes in particle size and PDI were 127.7 ± 1.3 nm and 0.227 ± 0.010, respectively. Finally, when 24 mg of TA was added, particle size and PDI were 113.8 ± 1.9 nm and 0.225 ± 0.011, respectively. As the amount of TA increased, the particle size decreased and the PDI increased. When 6 mg was added, there was no noticeable change, and aggregation occurred when 24 mg was added. Therefore, the amount of TA was determined as 12 mg (Figure 5).

### 3.4. Characterization of CUR-NSP and TA-CUR-NSP

The zeta potential of CUR-NSP and TA-CUR-NSP was measured by the ELS-Z2 (Osaka, Japan) in this study. Zeta potential values of CUR-NSP and TA-CUR-NSP were −35.55 ± 0.61 and −38.17 ± 2.40 mV, respectively. According to Müller et al., [34] a minimum zeta potential of 30 mV is required to exhibit good stability of nanosuspension; thus, TA-CUR-NSP was electrostatically stabilized.

**Figure 6:**
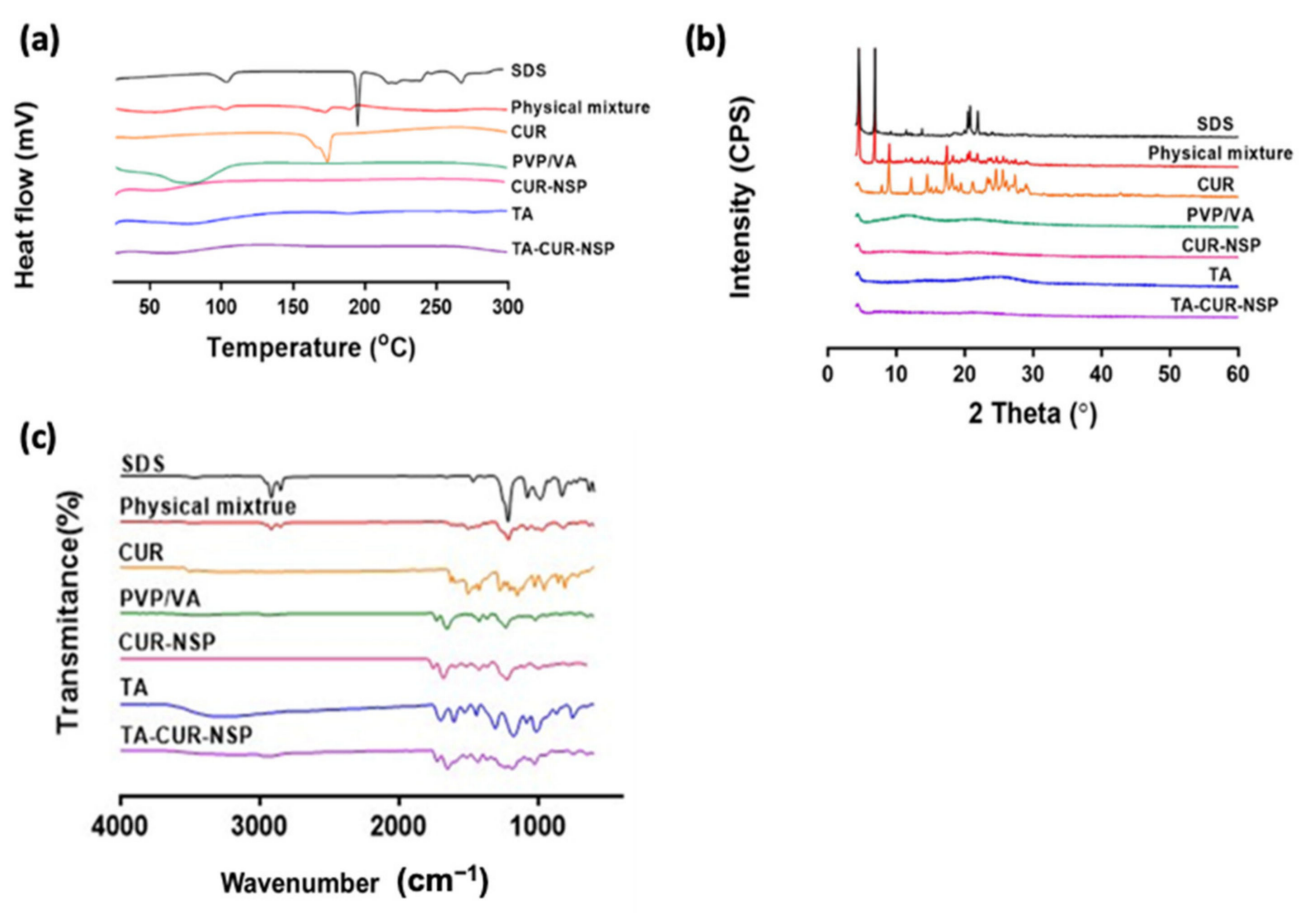
The physicochemical characterization of pure CUR, PVP/VA, SDS, CUR-NSP, physical mixture, TA and TA-CUR-NSP; (**a**) DSC thermograms; (**b**) PXRD patterns; and (**c**) FT-IR spectra.

DSC measurements were performed to investigate the crystallinity of CUR in CUR-NSP and TA-CUR-NSP. Figure 6a represents the DSC thermograms of pure CUR, SDS, PVP/VA, TA, CUR-NSP, TA-CUR-NSP, and physical mixture. In case of CUR, an endothermic peak was observed at 174 °C. Moreover, the thermal peak of SDS was detected at 198 °C, and no clear thermal peak of PVP/VA and TA were identified. The peak of CUR, PVA/VA, and SDS was also observed in the physical mixture, whereas TA-CUR-NSP did not show the peak of CUR, PVA/VA, and SDS. These results suggest that the CUR exists in amorphous and/or solubilized form in TA-CUR-NSP [35].

Figure 6b showed the XRD spectra of pure CUR, SDS, PVP/VA, TA, CUR-NSP, TA-CUR-NSP, and physical mixture. Numerous crystalline peaks could be identified in the pure CUR, and peaks appeared in the physical mixture at the following points (4.6°, 6.9°, 18.2°, 20.7°, 21.9°, and 23.9°). On the other hand, CUR-NSP and TA-CUR-NSP have peaks similar to those of PVP/VA and TA, respectively. These results also suggest that CUR exists in amorphous and/or solubilized form in NSPs [21].

The potential interaction between CUR and other materials was evaluated using FT-IR. The spectra of pure CUR, SDS, PVP/VA, TA, CUR-NSP, TA-CUR-NSP, and physical mixture were represented in Figure 6c. The FT-IR spectra of pure CUR exhibited 3505 cm^−1^ assigned to the phenolic O–H stretching. Additionally, there were sharp peaks at 1625 cm^−1^ (benzene ring of CUR), 1503 cm^−1^ (C=O of CUR), 1275 cm^−1^ (aromatic C–O stretching), and 1024 cm^−1^ (C–O–C stretching of CUR). In FT-IR, spectra of PVP/VA exhibited 1729 cm^−1^ (C=O stretching) and 1233 cm^−1^ (C–N stretching). SDS exhibited peaks of 2916 cm^−1^ (C–H stretching) and 1078 cm^−1^ (C–O stretching). In CUR-NSP, only overlapped peaks of CUR, PVP/VA, and SDS were observed, indicating no interaction between CUR and excipients. At the FT-IR spectra after being coated with TA, the formation of hydrogen bonds and the corresponding peak shift were observed. The O–H peak of TA shifted from 3225 to 2972 cm^−1^, indicating revealing the formation of hydrogen bonding between TA and CUR-NSP [36].

### 3.5. In Vitro Release Study

**Figure 7:**
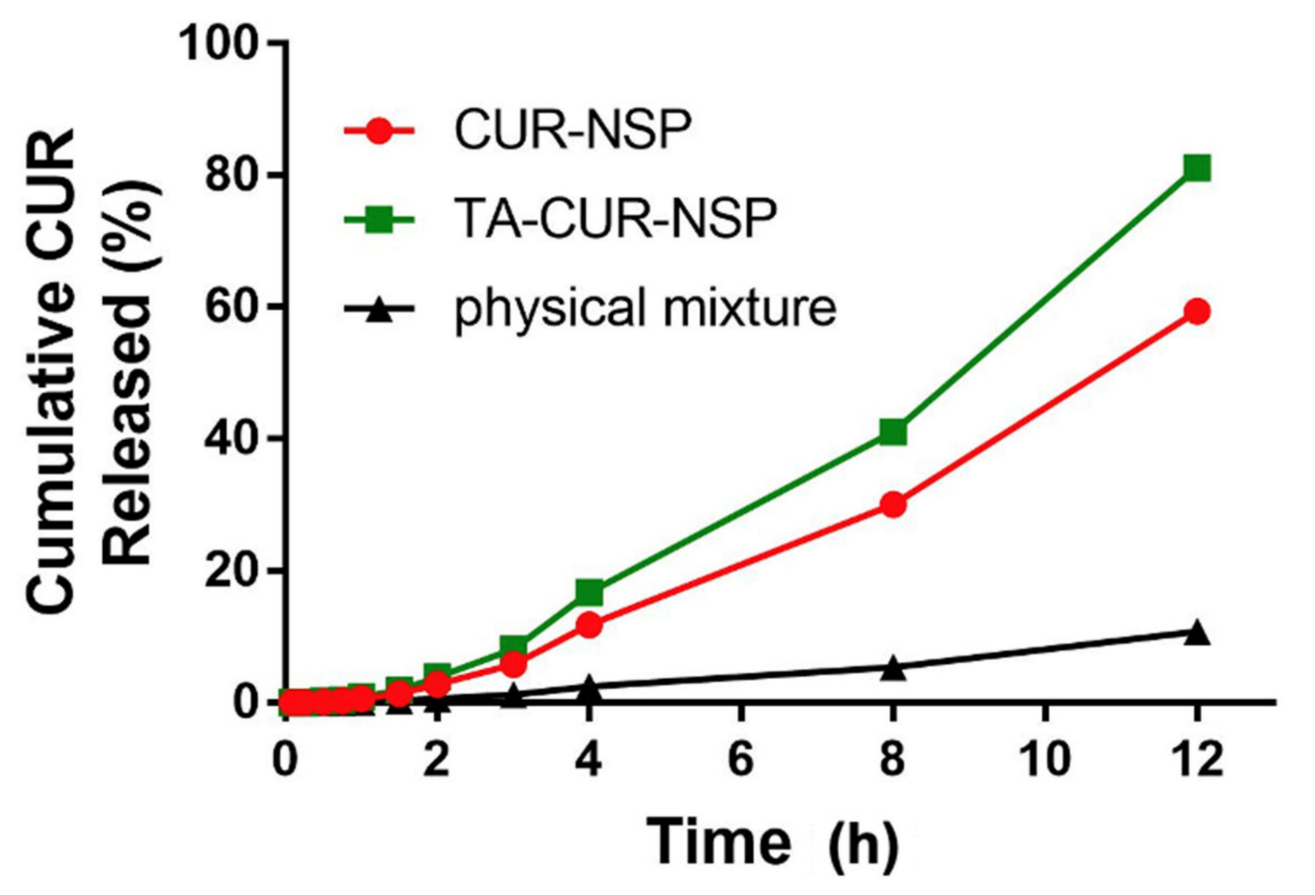
In vitro release profiles of CUR-NSP, TA-CUR-NSP, and physical mixture in PBS with 0.5% (*w*/*v*) (pH 7.4). Values are expressed as the mean ± SD (*n* = 3).

In order to determine the release pattern of CUR from NSPs, in vitro release tests were conducted in PBS (pH 7.4) with 0.5% (*w*/*v*) SDS. When nonionic surfactants such as Tween 80 or TPGS were used, aggregation occurred by reacting with TA. It was selected because aggregation did not occur only when SDS was used among several types of surfactants. Figure 7 shows the cumulative release of CUR from CUR-NSP, TA-CUR-NSP, and physical mixture. The composition of physical mixture was 30 mg of CUR, 150 mg of PVP/VA, 3 mg of SDS, and 12 mg of TA. The release of physical mixture showed about 10% at 12 h. By contrast, CUR release of TA-CUR-NSP and CUR-NSP was observed at 81.22 ± 0.83% and 59.41 ± 0.54% at 12 h, respectively. Obviously, the NSP formulation offered a moderate improvement in vitro release when compared with raw CUR, indicating that the solubility of CUR was improved after being prepared into the NSP. The reason for the higher release of TA-CUR-NSP compared with CUR-NSP is that TA improves the solubility of poorly soluble drugs and has an excellent stabilizing effect [37].

### 3.6. Mucoadhesion Aassay

**Figure 8:**
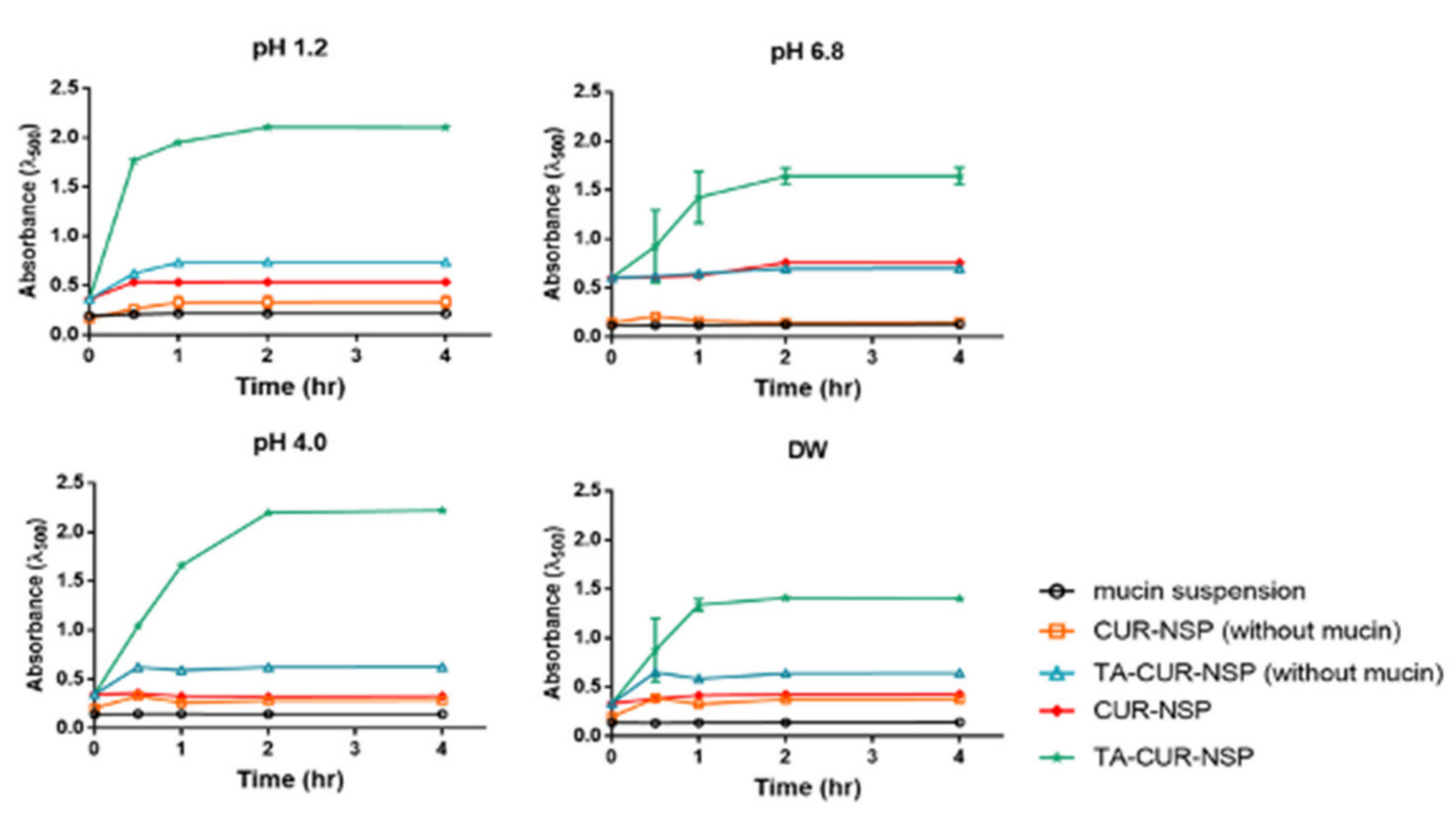
Mucoadhesion of TA-CUR-NSP and CUR-NSP with different pH values (1.2, 4.0, 6.8, and DW) of mucin at 37 °C.

Mucin is the main component of mucus covering the intestinal epithelium and serves as the first barrier for oral administration [38]. Therefore, we evaluated the affinity of NSPs to the mucous layer through experiments that confirmed the interaction of NSPs with mucin. In this study, changes in turbidity were observed to evaluate the adhesion of NSP and mucin. In addition, turbidity was measured in terms of absorbance. Moreover, four different pH values (pH 1.2, 4.0, 6.8, and DW) of mucus were selected to study the pH-dependent interaction of mucus with NSPs (Figure 8). Turbidity of CUR-NSP and TA-CUR-NSP in various pH conditions was stable for 4 h, indicating that CUR-NSP and TA-CUR-NSP are stable under various pH conditions. The mucin suspension showed constant turbidity regardless of the pH values. The turbidity changes according to the pH of each formulation increased until 30 min for both CUR-NSP and TA-CUR-NSP, but remained constant after 30 min. The turbidity of TA-CUR-NSP with mucin increased with time, regardless of the pH value of mucin suspension. On the other hand, there was no noticeable turbidity change in the mixture of CUR-NSP and mucin suspension. TA is a polyphenol molecule that binds strongly to various proteins. In general, when macromolecules such as TA or mucin are strongly entangled with each other, fine-sized randomly shaped particles are generated. So, the particle suspension becomes a turbid solution by the scatter of the visible light [39]. The mucoadhesion was confirmed through the interaction between TA-CUR-NSP and mucin. Moreover, improved mucoadhesion increases the retention time in the GI tract, which helps to increase local drug concentration and improve oral bioavailability [40].

### 3.7. Cell Studies

#### 3.7.1. Cytotoxicity Study

**Figure 9:**
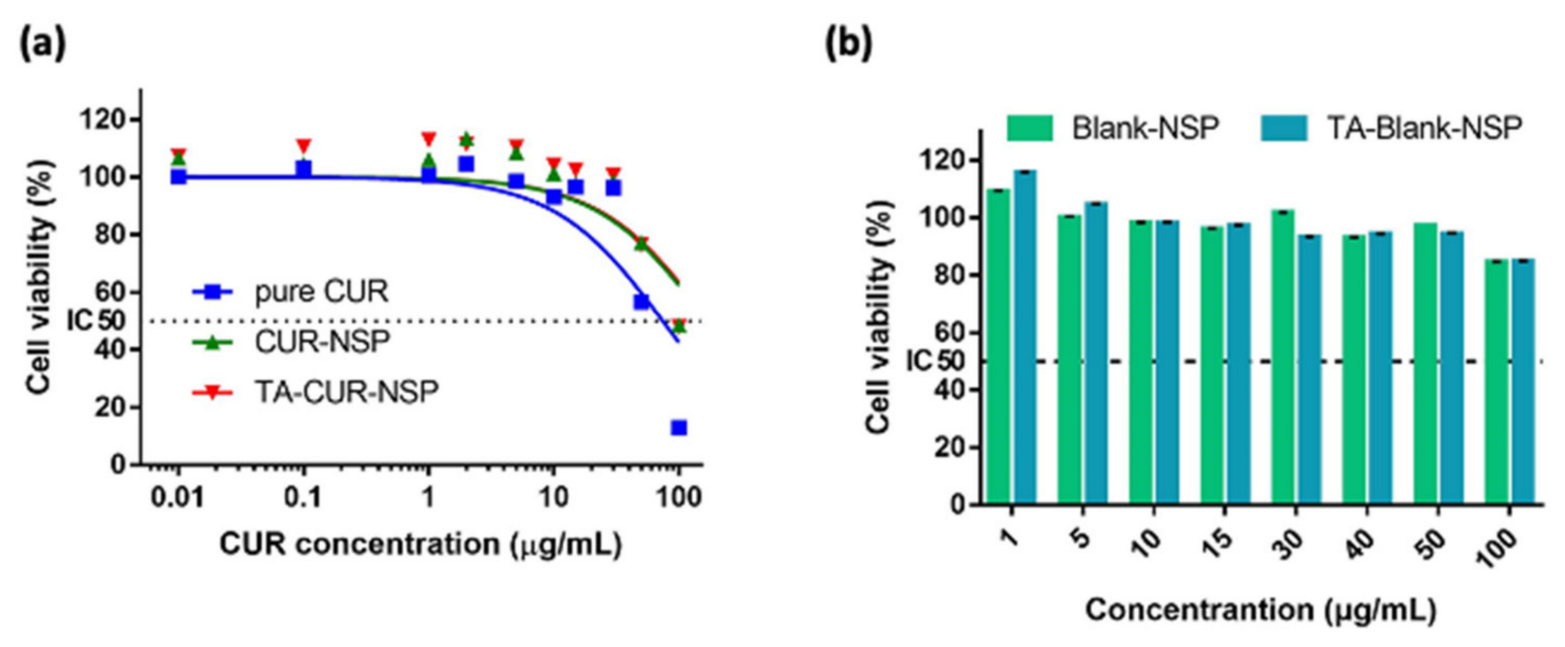
Cytotoxicity of CUR-NSP and TA-CUR-NSP. (**a**) Cytotoxicity of pure CUR, CUR-NSP, and TA-CUR-NSP; (**b**) Blank-NSP and TA-Blank-NSP on Caco-2 cells for 24 h. Values are expressed as the mean ± SD (*n* = 5).

The toxicity of the formulation in the GI tract was investigated by assessing cytotoxicity to Caco-2 cells. Differentiated Caco-2 cells are generally used to mimic the intestinal environment. Therefore, the cytotoxicity of formulations was evaluated against Caco-2 cell. According to the literature, above 70% of cell viability is considered non-toxic [33]. Figure 9a represented the cell viability of formulations containing CUR. IC_50_ values of CUR, CUR-NSP, and TA-CUR-NSP were 74.26, 165.5, and 173 µg/mL, respectively (0.20, 0.45, and 0.47 µM, respectively). For blank formulations, no cytotoxicity was found with a viability of less than 50% in the experimental concentration range (Figure 9b). These results indicated that the cytotoxicity was related to CUR concentrations of above 75 µg/mL (0.2 µM), not NSP formulation. However, in literature, the cytotoxic activity of TA has been reported against various tumor cell line. In this study, there is no statistical difference of cell viability between Blank NSP and Blank TA-NSP at the 100 µg/mL, which corresponds to the concentration of CUR. TA concentration of Blank TA-NSP at the 100 µg/mL, which corresponds to the concentration of CUR was around 400 µg/mL (~0.2 µM). In a previous study, IC_50_ of TA was reported to be about 46 µM against Caco-2 cell [41]. It indicates that the TA concentration of Blank TA-NSP was not enough to induce the cytotoxic activity of TA. In summary, TA-CUR-NSP may exhibit low cytotoxicity if compared with the CUR product. Thus, all formulations in this study are highly biocompatible and might be safe in the situation of oral administration. In this study, considering the exposure duration of TA-CUR-NSP to the intestinal tract, the cytotoxicity of TA-CUR-NSP was evaluated for 24 h. However, in the future, the systemic toxicity of TA-CUR-NSP should be assessed for clinical application.

#### 3.7.2. Cellular Uptake Study

**Figure 10:**
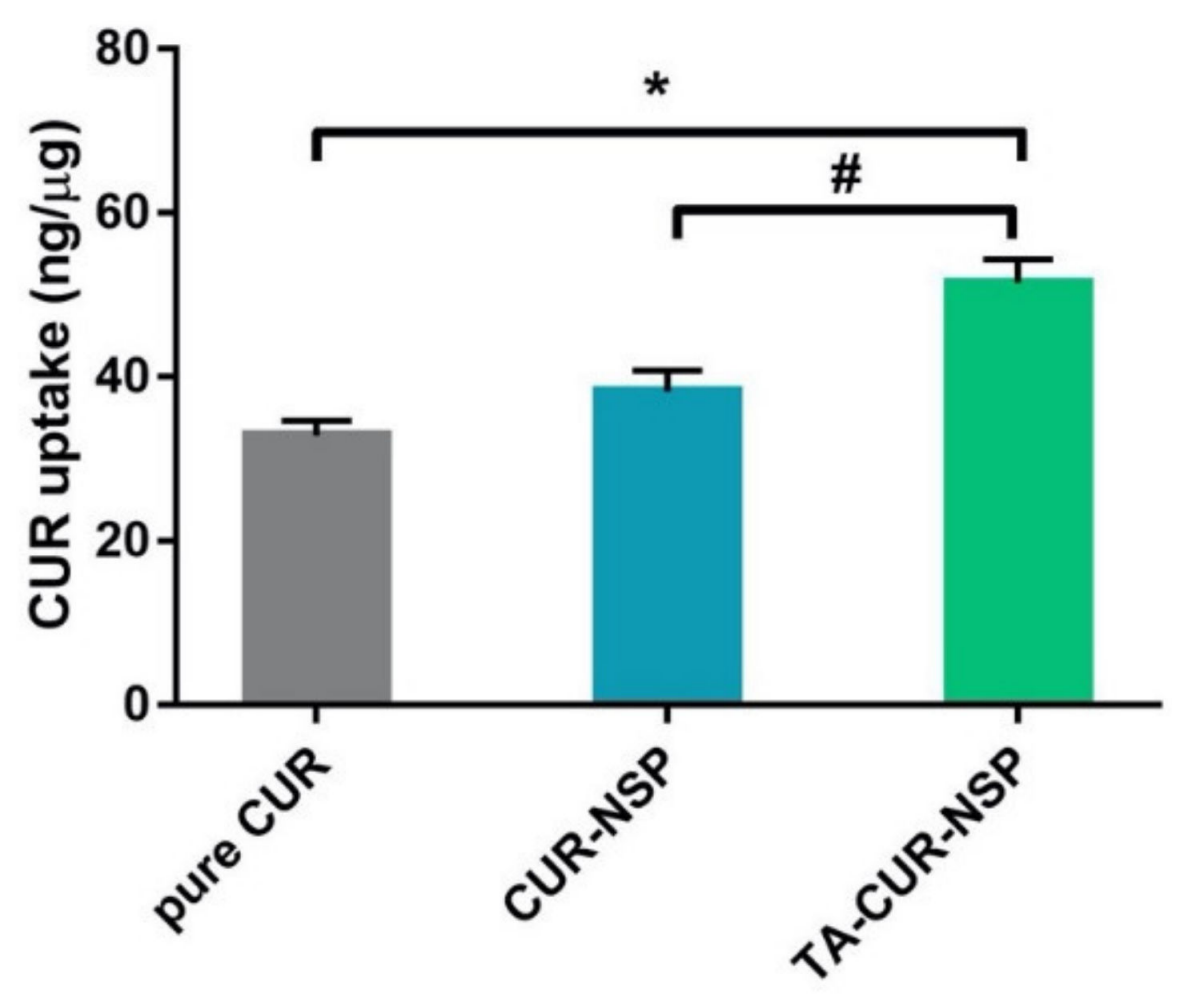
Cellular uptake of CUR, CUR-NSP, and TA-CUR-NSP into Caco-2 cells. The values are presented as mean ± SD (*n* = 3). * *p* < 0.05 versus CUR and # *p* < 0.05 versus CUR-NSP.

A cellular uptake study was carried out to investigate the effect of formulation on enhancing CUR uptake into Caco-2 cells. In Figure 10, the cellular uptake of TA-CUR-NSP (51.5 ng/µg) was increased by 1.6-fold compared with that of pure CUR (32.9 ng/µg), which was higher than CUR-NSP (38.2 ng/µg) (*p* < 0.05). These results indicated that cellular uptake of CUR was increased by TA-coated NSP formulation.

**Figure 11:**
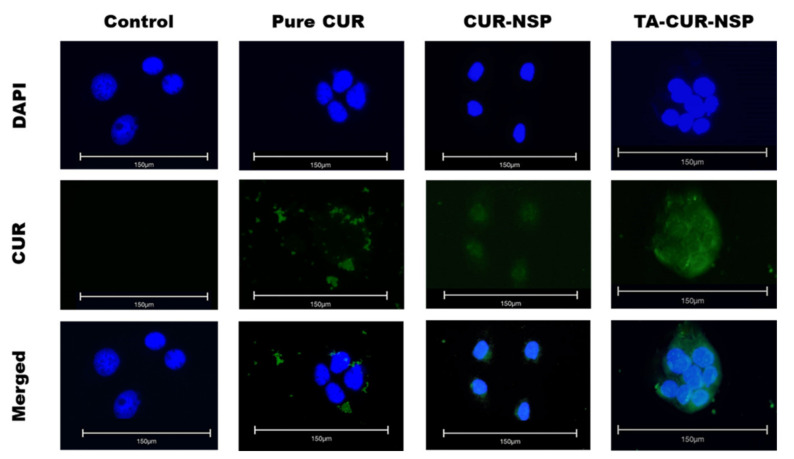
Cellular uptake study in Caco-2 cells observed with fluorescence microscope. The cells were incubated with CUR solution and formulations (30 μg/mL of CUR). Blue and green colors indicate DAPI staining and CUR, respectively. Scale bar is 150 μm.

Figure 11 is a fluorescence observation used to visually estimate the cellular uptake of CUR in Caco-2 cells. The fluorescence intensity of TA-CUR-NSP was stronger than that of the CUR solution and CUR-NSP, indicating that TA-coated formulation can increase the cellular uptake or accumulation of CUR. The reason was that enriched amount of CUR observed in the cellular compartment with TA-CUR-NSP was due to the presence of TA, which was reported to inhibit P-gp function by partially inhibiting ATPase [42]. According to the literature, the structural features of polyphenols are required in ATPase activity of P-gp; in particular, structural features such as 5-OH, 5-OCH3, 6-OH, 7-OCH3, 3′-OH, and 4′-OH are essential for inhibition on P-gp [43]. The TA used in this study contains the galloyl group, which consists of the hydroxyl group at the 5′ site. Moreover, it has been reported that the TA inhibits P-gp function by partially inhibiting ATPase. Thus, the P-gp inhibition effect of TA could contribute to the enhanced cellular uptake effect of TA-CUR-NSP. Moreover, CUR has been known as the modulator of P-gp function. In several studies, CUR inhibits not only the P-gp function but also the expression of P-gp at the mRNA level. Thus, TA-CUR-NSP could potentiate the cell permeability (cellular intake) of CUR.

In the cellular uptake study, TA-CUR-NSP showed the highest uptake if compared with CUR and CUR-NSP. It indicates that the intracellular CUR concentration of TA-CUR-NSP was higher than those of CUR and CUR-NSP. In the literature, the cytotoxicity of CUR was increased with increasing uptake of CUR [44]. However, in this study, TA-CUR-NSP had the lowest cytotoxicity. It might be due to the difference in the CUR release rate. CUR of CUR-NSP and TA-CUR-NSP was encapsulated or stabilized by surfactant and polymer. In in vitro release study, around 50–80% of CUR was released from CUR-NSP and TA-CUR-NSP at 12h. Thus, it indicates that the CUR was localized in the cell as the encapsulated form and the CUR was released for a prolonged period.

### 3.8. Antioxidant Activity

**Figure 12:**
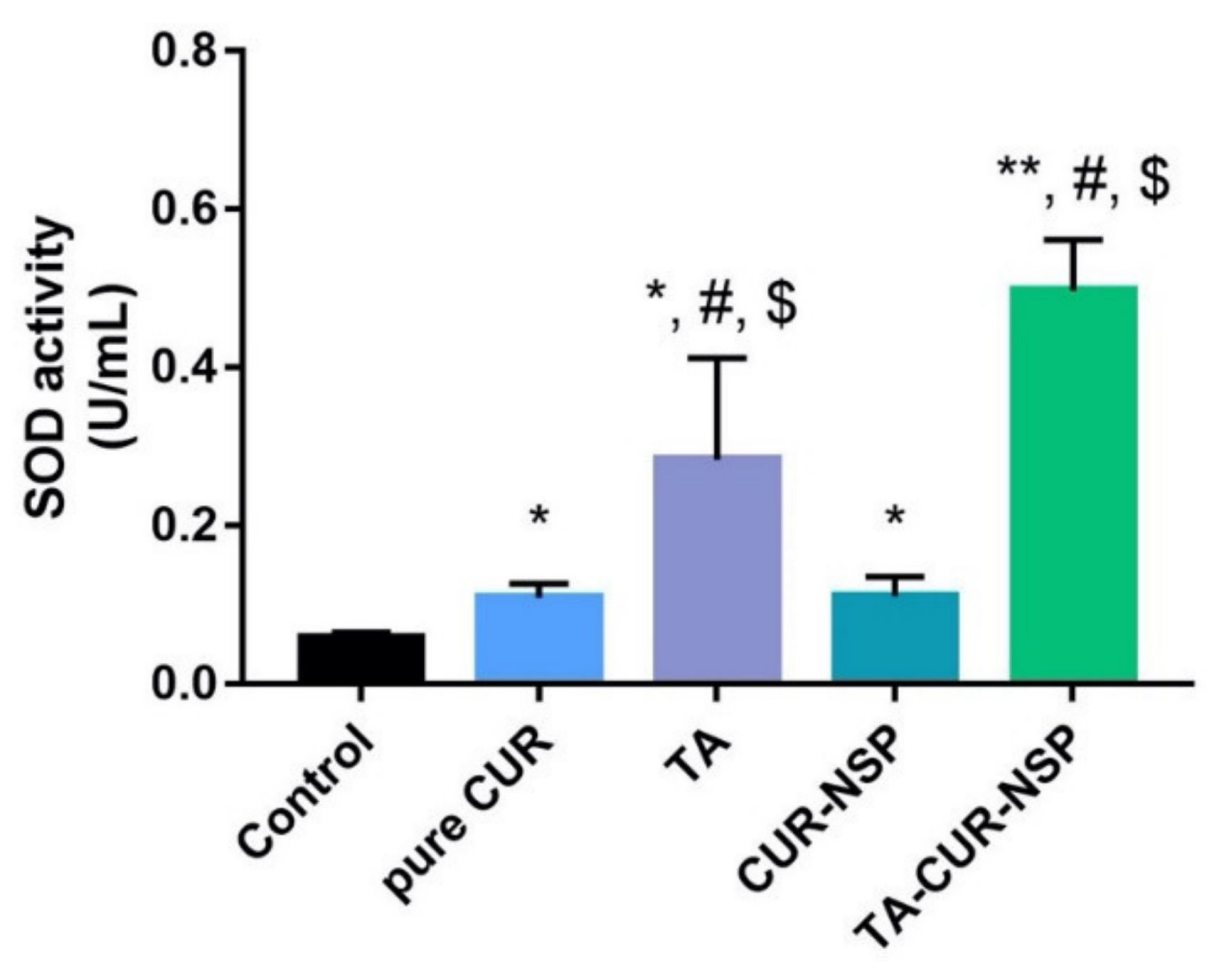
The SOD activity of control (cell medium), pure CUR, CUR-NSP, and TA-CUR-NSP in Caco-2 cells. The cells incubated CUR formulation equivalent to 30 μg/mL and then induced oxidative stress by 500 μM H_2_O_2_. The values are presented as mean ± SD (*n* = 3). * *p* < 0.05 versus control, ** *p* < 0.05 versus control, # *p* < 0.05 versus pure CUR, and $ *p* < 0.05 versus CUR-NSP.

The antioxidant activity was studied after Caco-2 cells were pre-incubated with the different formulation (cell medium, pure CUR, TA, CUR-NSP, and TA-CUR-NSP) preparations for 24 h and then treated with H_2_O_2_ for induced oxidative stress after 24 h (Figure 12). Interestingly, TA-CUR-NSP showed significantly high SOD activity. Similar levels of SOD activity were observed for pure CUR and CUR-NSP. The SOD activity of TA was observed higher than pure CUR and CUR-NSP. These results can be explained for two reasons. The first is because TA-CUR-NSP had a good uptake in Caco-2 cells compared with other formulations, as shown in the previous cellular uptake study. Second, because TA also has an antioxidant activity as a polyphenol [45].

### 3.9. Pharmacokinetics Study

**Figure 13:**
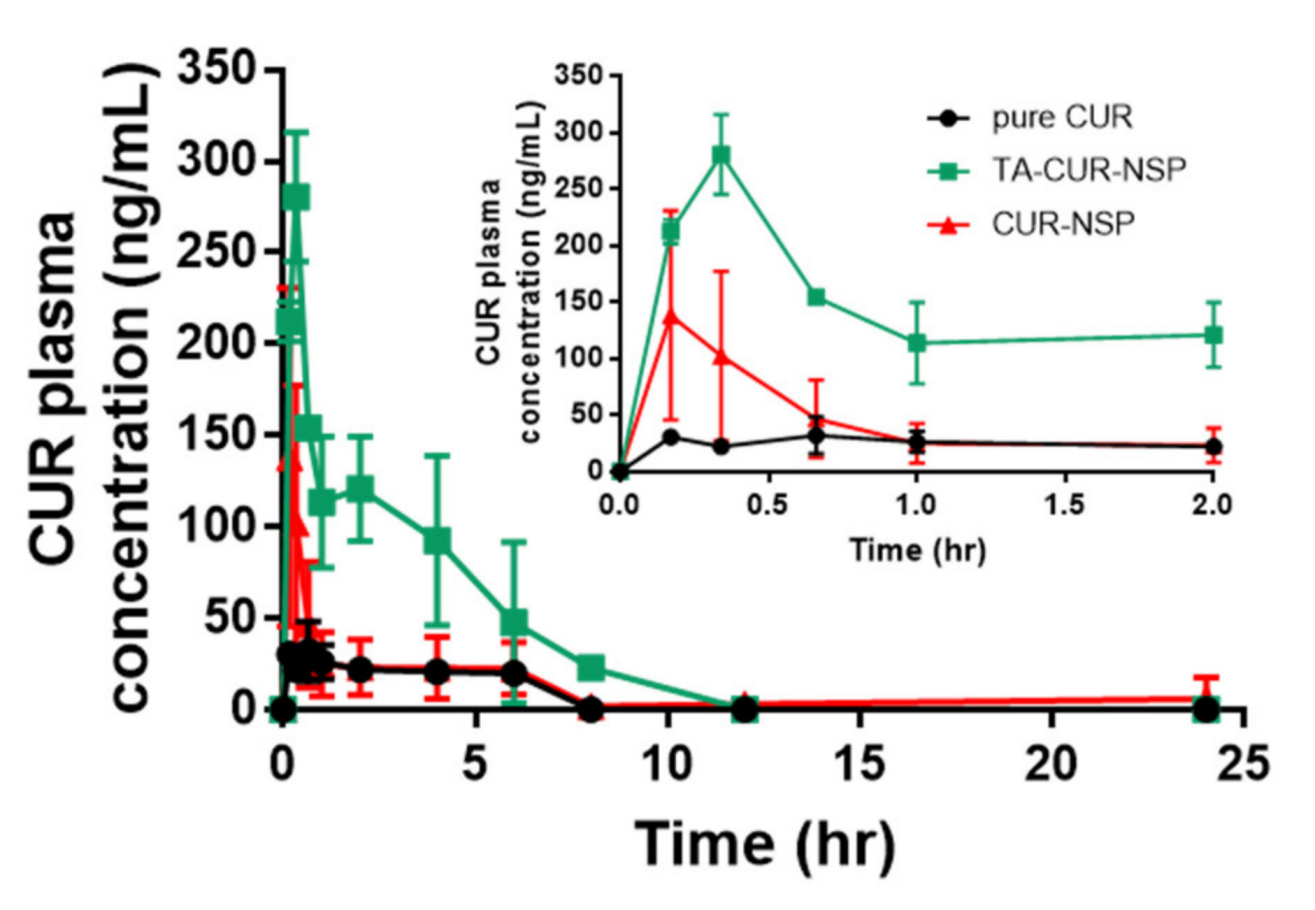
Plasma concentration-time curve of CUR in rats after single oral administration of pure CUR, CUR-NSP, and TA-CUR-NSP at a dose equivalent to 35 mg/kg CUR. Values are expressed as the mean ± SD (*n* = 5).

In cell and mucoadhesion studies, it was confirmed that CUR particles of TA-CUR-NSP had better cellular uptake and higher affinity for mucous membranes than pure CUR and CUR-NSP. Figure 13 shows the plasma concentration of CUR after the administration of pure CUR, CUR-NSP, and TA-CUR-NSP. Plasma concertation of TA-CUR-NSP was superior to that of pure CUR and CUR-NSP. It indicates that TA-CUR-NSP enhanced the oral absorption of CUR. Pharmacokinetic parameters are listed in Table 5. Parameters such as C_max_ and AUC_0–24_ reflect the rate and extent of drug absorption. In this study, C_max_ value of TA-CUR-NSP were 1.5-fold and 7.2-fold higher than those of CUR-NSP and pure CUR, respectively (*p* < 0.05). Moreover, AUC_0–24_ of TA-CUR-NSP was 5-fold and 2.9-fold higher than that of CUR-NSP and pure CUR, respectively. These results indicate that pharmaceutical technologies including TA-coated NSP formulations have supported a significant increase in the bioavailability of CUR. This increase in bioavailability is explained for several reasons. Firstly, nanoparticles are easier to pass through cell membranes in organs and get interacted rapidly with biological systems, so nanosized delivery systems will probably be the most appropriate for highly hydrophobic agents such as CUR [46]. Secondly, due to P-gp inhibitory function by TA, CUR particles prolonged intestinal retention and improved trans-epithelial transport properties [47]. Finally, nanometer-sized drug particles increase the total effective surface area, thereby improving the dissolution rate, and increasing the concentration gradient due to the decrease in the thickness of the diffusion layer surrounding the drug particles [48]. Due to these processes, TA-CUR-NSP increased the bioavailability of CUR. TA is the natural polyphenol, which interacts with biopolymers such as mucin, collagen, gelatin, and albumin through noncovalent interactions. TA-CUR-NSP could increase the mucoadhesion of NSP to mucus layer through noncovalent interactions. When TA-CUR-NSP is orally administered, the hydrogen bond between TA and CUR-NSP would be hydrolyzed. After that, CUR-NSP would be absorbed into the epithelial cell. Thus, the increase in CUR absorption might be due to the increase in TA-CUR-NSP residence time. However, for the clinical application of TA-CUR-NSP, absolute bioavailability should be evaluated in further study.

To draw the whole picture of CUR-NSP metabolism, the amount of conjugated CUR metabolites (glucuronide or sulfate) should be evaluated. Moreover, various studies about the evaluation of polyphenol’s metabolites have been reported [49,50]. Thus, to reveal the metabolism of CUR-NSP, the pharmacokinetic evaluation of CUR metabolites (conjugated or reductive metabolites) should be evaluated in the future.

TA-CUR-NSP was successfully TA-coated on the optimized NSP, which improved solubility and oral bioavailability. TA-CUR-NSP with particle size of 127.7 ± 1.3 nm and low precipitation of 1.5% could be obtained. TA-CUR-NSP had a synergistic effect on oral absorption through enhanced release and mucoadhesion. The pharmacokinetic study in rats exposed enhancement of oral bioavailability of CUR by 5-fold and 2.9-fold compared with pure CUR and CUR-NSP. In conclusion, we discovered the therapeutic potential of TA-CUR-NSP for improving oral absorption of CUR. In addition, the TA-coated formulation could be a promising technique to overcome the low bioavailability of drugs.

## 4. Conclusions

TA-CUR-NSP was successfully TA-coated on the optimized NSP, which improved solubility and oral bioavailability. TA-CUR-NSP with particle size of 127.7 ± 1.3 nm and low precipitation of 1.5% could be obtained. In the characterization and evaluation studies, TA-CUR-NSP had a synergistic effect on oral absorption through enhanced release and mucoadhesion. The pharmacokinetic study in rats exposed enhancement of oral bioavailability of CUR by 5-fold and 2.9-fold compared with pure CUR and CUR-NSP. In conclusion, we discovered the therapeutic potential of TA-CUR-NSP for improving oral absorption of CUR. Additionally, the TA-coated formulation could be a promising technique to overcome the low bioavailability of drugs.

## Figures and Tables

**Table 1:** Factors and responses of the Box–Behnken design for CUR-NSP.

Factors	Range	
	Low Limit	High Limit
X_1_: SDS amount (mg)	0	5
X_2_: PVP/VA amount (mg)	150	250
X_3_: Volume of aqueous phase (mL)	5	15
Responses	Goal	
Y_1_: Particle size (nm)	Minimize	
Y_2_: Polydispersity index (PDI)	Minimize	
Y_3_: Precipitation (%)	Minimize	

**Table 2:** The composition of CUR-NSP and observed responses. Values are presented as mean ± SD (*n* = 3).

Run	Factors			Responses		
	X_1_	X_2_	X_3_	Y_1_	Y_2_	Y_3_
	SDS (mg)	PVP/VA (mg)	Volume (mL)	Particle Size (nm)	PDI (-)	Precipitation (%)
1	2.5	200	10	189.2 ± 1.9	0.106 ± 0.021	5.59 ± 0.10
2	5	200	5	209.0 ± 2.6	0.173 ± 0.014	8.70 ± 0.06
3	0	200	15	215.1 ± 1.6	0.081 ± 0.007	6.10 ± 1.56
4	2.5	200	10	186.3 ± 1.3	0.094 ± 0.007	5.49 ± 0.48
5	2.5	200	10	185.2 ± 2.2	0.095 ± 0.007	4.37 ± 1.44
6	2.5	250	15	180.4 ± 0.8	0.125 ± 0.123	2.74 ± 0.83
7	0	200	5	253.1 ± 1.7	0.083 ± 0.010	16.70 ± 3.33
8	5	200	15	145.3 ± 0.8	0.181 ± 0.023	2.34 ± 2.87
9	2.5	150	5	197.5 ± 1.4	0.090 ± 0.014	6.87 ± 5.50
10	2.5	200	10	182.6 ± 1.6	0.089 ± 0.015	4.07 ± 0.62
11	2.5	150	15	151.8 ± 0.7	0.114 ± 0.007	2.51 ± 0.42
12	2.5	200	10	193.1 ± 2.4	0.096 ± 0.012	5.28 ± 3.14
13	2.5	250	5	251.6 ± 1.2	0.108 ± 0.010	14.00 ± 1.85
14	0	250	10	231.3 ± 1.2	0.080 ± 0.012	9.42 ± 2.80
15	0	150	10	192.8 ± 0.2	0.084 ± 0.012	5.45 ± 2.00
16	5	150	10	133.4 ± 3.3	0.170 ± 0.009	1.51 ± 0.91
17	5	250	10	181.1 ± 2.3	0.195 ± 0.009	3.40 ± 1.84

**Table 3:** Statistical models and parameters.

Response	Suggested Model	*p*-Value	Lack of Fit *p*-Value	R^2^	Adjusted R^2^	Predicted R^2^	Adequate Precision
$Y_{1}$: Particle size (nm)	Quadratic	0.0003	0.6117	0.9944	0.9871	0.9640	42.36
$Y_{2}$: Polydispersity index	Quadratic	0.0003	0.3374	0.9864	0.9689	0.8737	22.28
$Y_{3}$: Precipitation (%)	Quadratic	0.0003	0.6319	0.9895	0.9759	0.9346	31.18

**Table 4:** Predicted and observed values of optimal CUR-NSP. Values are presented as mean ± SD (*n* = 3).

Optimal Factors	Response	95% CI Low Predicted Value	Predicted Value	95% CI High Predicted Value	Observed Value	Error Percentage (%)
X_1_: 3 mg	$Y_{1}$: Particle size (nm)	141.77	147.4	153.02	146.1 ± 0.8	8.2
X_2_: 150 mg	$Y_{2}$: Polydispersity index	0.1	0.11	0.12	0.108 ± 0.015	2.3
X_3_: 12 mL	$Y_{3}$: Precipitation (%)	0.55	1.51	2.47	1.55 ± 1.09	0.9

**Table 5:** Pharmacokinetic parameters of CUR after single oral administration of pure CUR, CUR-NSP, and TA-CUR-NSP at a dose equivalent to 35 mg/kg CUR. Values are expressed as the mean ± SD (*n* = 5). ^*^ *p* < 0.05 versus pure CUR and ^#^ *p* < 0.05 versus CUR.

Pharmacokinetic Parameters	Formulations		
	Pure CUR	CUR-NSP	TA-CUR-NSP
T_max_ (h)	0.61 ± 0.42	0.17 ± 0.00	0.34 ± 0.00
C_max_ (ng/mL)	39.07 ± 10.37	183.98 ± 12.75	280.43 ± 35.32 ^*,#^
AUC_0–24_ (ng∙h/mL)	152.95 ± 17.30	267.56 ± 55.43	763.13 ± 199.73 ^*,#^
T_1/2_ (h)	1.86 ± 0.61	2.46 ± 0.12	4.71 ± 1.56
Relative bioavailability	-	174%	499%

## Data Availability

Not applicable.
